# Lower Vitamin D Metabolites Levels Were Associated With Increased Coronary Artery Diseases in Type 2 Diabetes Patients in India

**DOI:** 10.1038/srep37593

**Published:** 2016-11-24

**Authors:** Ramu Adela, Roshan M Borkar, Murali Mohan Bhandi, Gayatri Vishwakarma, P. Naveen Chander Reddy, R. Srinivas, Sanjay K Banerjee

**Affiliations:** 1 Drug Discovery Research Center, Translational Health Science and Technology Institute (THSTI), Faridabad, HR-121001 (RA, SKB), India; 2 National Center for Mass Spectrometry, Indian Institute of Chemical Technology (CSIR-IICT), Hyderabad (RMB, MMB, RS), India; 3 Clinical Development Service Agency (CDSA), Translational Health Science and Technology Institute (THSTI), Faridabad, HR-121001 (GV), India; 4 Cardiology Division, Mediciti Hospitals, Hyderabad-500063 (PNCR), India

## Abstract

The purpose of the present study was to measure six vitamin D metabolites and to find the association between vitamin D deficiency and coronary artery diseases in diabetes (T2DM_CAD). Four groups [control (n = 50), type 2 diabetes (T2DM, n = 71), coronary artery diseases (CAD, n = 28), T2DM_CAD (n = 38)] of total 187 subjects were included in the study. Six vitamin D metabolites (D_2_, D_3_, 25(OH)D_2_, 25(OH)D_3_, 1,25(OH)_2_D_2_, 1,25(OH)_2_D_3_), total 25(OH)D and total 1,25(OH)_2_D were measured by UPLC/APCI/HRMS method in these subjects. Although all the vitamin D metabolites were significantly decreased in T2DM_CAD as compared to both control and T2DM subjects (p < 0.05), only two metabolites i.e., 25(OH)D_3_ and total 25(OH)D were significantly (p < 0.05) decreased in the T2DM subjects as compared with the control subjects (p < 0.05). Vitamin D_3_, 1,25(OH)_2_D_2_, 25(OH)D, and 1,25(OH)_2_D levels were significantly decreased in T2DM_CAD subjects as compared with CAD subjects (p < 0.05). Further, multiple logistic regression analysis revealed that total 25(OH)D and total 1,25(OH)_2_D can be used to predict T2DM (OR 0.82.95% CI 0.68–0.99; p = 0.0208) and T2DM with CAD (OR 0.460, 95% CI 0.242–0.874; p = 0.0177), respectively. Our data concludes that lower concentration of 1,25(OH)_2_D is associated with type 2 diabetes coexisting with coronary artery diseases in South Indian subjects.

Type 2 diabetes is characterized by hyperglycemia as well as insulin resistance. It is a complex metabolic disease accompanied by several complications such as obesity, hypertension, dyslipidemia and coronary artery disease (CAD). All these complications are interlinked and not understood completely^1^. As the disease progresses, diabetic patients develop micro and macro vascular complications. Hyperglycemic condition and insulin resistance in adults increases the risk of heart attack, stroke, angina, and coronary artery disease. Cardiovascular risk is two to four fold higher in diabetic subjects as compared to non-diabetic subjects^2^,^3^,^4^. Several factors like obesity, hyperglycemia, hypertension, inflammation, endothelial dysfunction and oxidative stress are responsible for the increased risk of cardiovascular complication among diabetic patients. Among all factors, lower vitamin D level in blood is regarded as one of the important risk factor to develop cardiovascular complication.

Vitamin D is a secosteroid that exists in two forms i.e., ergocalciferol (D_2_) and cholecalciferol (D_3_). Ergocalciferol (D_2_) is synthesized from the vegetable sources while cholecalciferol (D_3_) is either synthesized from the epidermis by exposure to the UV radiation (sunlight) or available from oily fish supplementation. Vitamin D (D_2_ and D_3_) is converted into its active metabolite 1,25(OH)_2_D by the two hydroxylation steps. Vitamin D (D_2_ and D_3_) are hydroxylated by the liver and converted into 25 hydroxy D [25(OH)D_2_ and 25(OH)D_3_]. These two were further hydroxylated into two active metabolites, 1,25(OH)_2_D [1,25(OH)_2_D_2_ and 1,25(OH)_2_D_3_] in the kidney. These active metabolites bind with the vitamin D receptor and exert its function^5^ (Fig. 1). Vitamin D receptors are present in many cells like pancreatic β cells, cardiomyocytes, endothelial cells and vascular smooth muscle cells. Vitamin D plays pivotal role in the bone and mineral metabolism. Vitamin D deficiency is a common health problem worldwide and is the cause for osteoporosis and osteomalacia, rickets and other bone related disorders. In the last decade, researchers observed that lower vitamin D levels were associated with metabolic diseases like type 1 diabetes, obesity, insulin resistance, cardiovascular diseases, and cancer^6^,^7^,^8^. Many studies reported that Indian subjects are more prone to vitamin D deficiency despite the availability of abundant sunshine throughout the year in many parts of India^9^,^10^,^11^,^12^,^13^,^14^,^15^,^16^,^17^. Although, there are some studies from Indian population but none of these studies looked into the association of vitamin D metabolites with coronary artery diseases coexisting with type 2 diabetes. For last few decades more than 50 metabolites of vitamin D have been described^18^. Among them very few have been quantified in blood.

Currently, general awareness of association of vitamin D insufficiency and increased risk of several diseases has been increased. However, it is not clear which vitamin D metabolite should be quantified and what is the association of each metabolite with increased risk of diseases. Among all vitamin D metabolites, only 25(OH)D and 1,25(OH)_2_D have received great attention. Hence in the present study six vitamin D metabolites have been quantified by UPLC/APCI/HRMS method. Therefore, the purpose of the present study was to measure six vitamin D metabolites, total 25(OH)D and total 1,25(OH)_2_D levels in control, T2DM, CAD and T2DM with CAD and to find the association between vitamin D deficiency and coronary artery diseases in diabetes.

## Results

Four groups (Control, T2DM, CAD and T2DM_CAD) of total 187 subjects were included in this present study. The serum level of total 25(OH)D was well classified in the previous literature^19^,^20^. Anthropometric and clinical characteristics of all subjects based on 25(OH)D categories were presented in Table 1. There were no significant differences in age, body mass index (BMI), systolic and diastolic blood pressure (BP), creatinine, estimated glomerular filtration rate (eGFR), uric acid, creatinine kinase myoglobin isoenzyme (CK-MB), and apolipoprotein B (Apo-B). Glycated hemoglobin (HbA1c) levels were increased significantly (p < 0.05) in patients having 20–30 ng/ml and <20 ng/ml at 25(OH)D levels as compared with subjects having >30 ng/ml of 25(OH)D. Similarly, fasting blood sugar (FBS) levels were also increased in patient groups having 25(OH)D levels 20–30 ng/ml and <20 ng/ml as compared with patients having >30 ng/ml.

Among all T2DM subjects, 43 subjects had history of hypertension. Among CAD subjects, 23 subjects were diagnosed with acute myocardial infarction (MI) and five were with unstable angina. Among T2DM_CAD subjects, 31 subjects were diagnosed with acute myocardial infraction and 7 were diagnosed with unstable angina.

### Distribution of vitamin D_2_ and D_3_ metabolites in control, T2DM, CAD and T2DM_CAD groups

**Figure 2:**
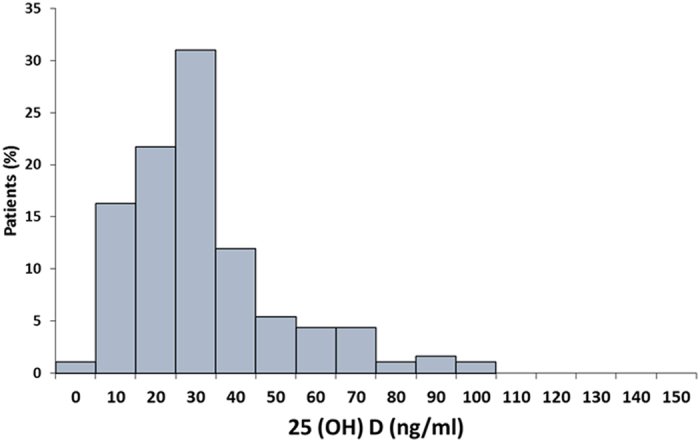
Distribution of serum 25(OH)D concentrations in total subjects.

Distribution of total 25(OH)D levels in all subjects was mentioned in Fig. 2. The median total 25(OH)D concentrations in the total subjects was 32.3 (range 7.9–99.0). According to the previous literature, serum total 25(OH)D concentrations were well categorized as sufficient >30 ng/ml, insufficient 30–20 ng/ml and deficient <20 ng/ml^20^. However, no such categories were established for the other metabolites. In present study, cut off points was set based on the median of the individual metabolites in total subjects. Cutoff values for all three 25(OH)D_3_, Vitamin D_2_ and total 25(OH)D metabolites were categorized as >30, 30–20, and <20 ng/ml. Other vitamin D metabolites like vitamin D_3_ (>20 and <20 ng/ml), 1,25(OH)_2_D_3_ (>6 and <6 ng/ml), 25(OH)D_2_ (>4 and <4 ng /ml), 1,25(OH)_2_D_2_ (>4 and <4 ng/ml) and 1,25(OH)_2_D (>11 and <11 ng/ml) were categorized as stated to find distribution of patients in different groups.

**Figure 3:**
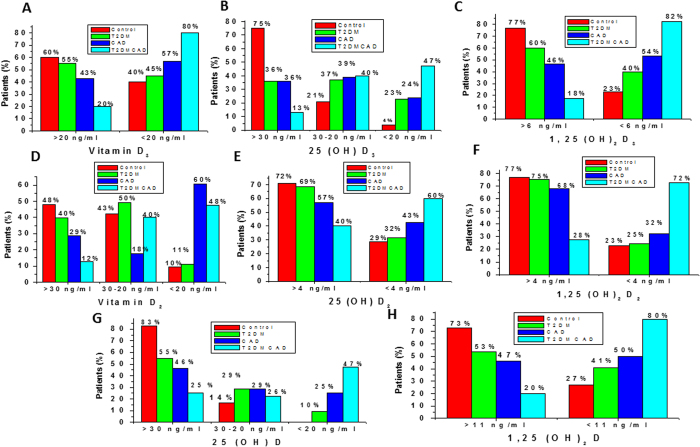
Distribution of vitamin D_3_, Vitamin D_2_ and 25(OH)D and 1,25(OH)2D metabolites in control, T2DM, CAD and T2DM_CAD. (**A**) Distribution of Vitamin D_3_ levels according to serum concentration i.e., <20 ng/ml verses >20 ng/ml. (**B**) Distribution of 25(OH)D_3_ levels according to serum concentrations i.e., >30 ng/ml, 30–20 ng/ml and <20 ng/ml. (**C**) Distribution of 1,25(OH)_2_D_3_ levels according to serum concentrations i.e., >6 ng/ml verses <6 ng/ml. (**D**) Distribution of vitamin D2 levels according to serum concentrations i.e., >30 ng/ml, 30–20 ng/ml and <20 ng/ml. (**E**) Distribution of 25(OH)D2 levels according to serum concentrations i.e., >4 ng/ml verses <4 ng/ml. (**F**) Distribution of 1,25(OH)_2_D2 levels according to serum concentration i.e., >4 ng/ml verses <4 ng/ml. (**G**) Distribution of 25(OH)D levels according to serum concentrations i.e., >30 ng/ml, 20–30 ng/ml and <20 ng/ml. (**H**). Distribution of 1,25(OH)_2_D levels according to serum concentrations i.e., >11 ng/ml verses <11 ng/ml.

In the present study, higher percentage of subjects having optimum or higher level of vitamin D_3_ (>20 ng/ml), 25(OH)D_3_ (>30 ng/ml) and 1,25(OH)_2_D_3_ (>6 ng/ml) metabolites were observed in control group. However, the percentage of diabetes along with CAD subjects were increased when we considered lower concentration of vitamin D3 (<20 ng/ml), 25(OH)D_3_ (<20 ng/ml) and 1,25(OH)_2_D_3_ (<6 ng/ml) metabolites. Lower percentage of T2DM, CAD and T2DM with CAD subjects were observed when we considered higher concentration of vitamin D_3_ metabolites. However, the percentage has been increased when we consider lower concentration of vitamin D_3_ metabolites (Fig. 3A,B and C). Similar changes were also observed for all three vitamin D_2_ metabolites (Vitamin D_2_, 25(OH)D_2_, 1, 25(OH)_2_D_2_) (Fig. 3D,E and F).

### Distribution of total 25(OH)D and total 1,25(OH)_2_D in control, T2DM, CAD and T2DM_CAD subjects

In the present study, higher percentage of subjects having optimum levels of total 25(OH)D (>30 ng/ml) and total 1,25(OH)_2_D (>11 ng/ml) were observed in control subjects. However, the percentage of diabetes along with CAD subjects (47% and 80%) were increased in subjects having lower concentration of total 25(OH)D_3_ (<20 ng/ml) and total 1,25(OH)_2_D (<11 ng/ml) when compared with control subjects (25% and 20%) (Fig. 3G and H).

### Vitamin D metabolites levels in control, T2DM and T2DM_CAD

**Figure 4:**
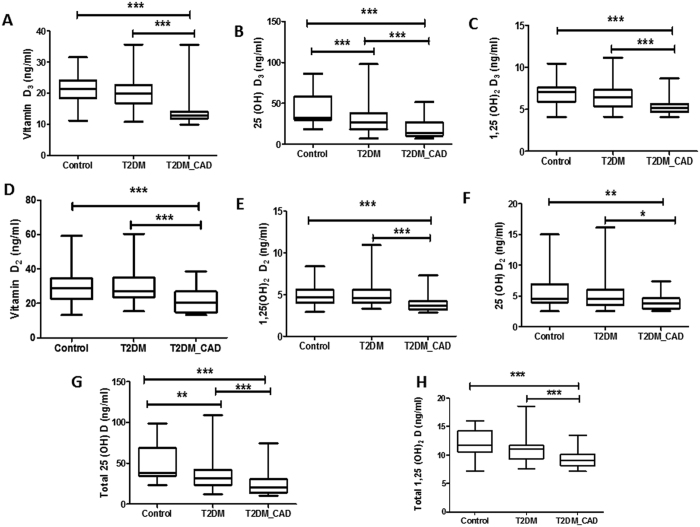
Serum vitamin D metabolites levels in control, T2DM and T2DM_CAD groups. Serum levels of vitamin D_3_ (**A**), 25(OH)D_3_ (**B**), 1,25(OH)_2_D_3_ (**C**)vitamin D_2_ (**D**), 25(OH)D_2_ (**E**), 1,25(OH)_2_D_2_ (**F**) 25(OH)D (**G**), and 1,25(OH)_2_D (**H**) were determined in control, T2DM, and T2DM_CAD groups. Data were expressed as median and interquartile range. p < 0.05 was considered significant.

All six vitamin D metabolites and total 25(OH)D, total 1,25(OH)_2_D were significantly lowered in T2DM_CAD group as compared to control group (p < 0.05) (Fig. 4A–H). Similarly, all six vitamin D metabolites levels and total 25(OH)D, total 1,25(OH)_2_D were significantly (p < 0.05) decreased in T2DM_CAD group as compared to T2DM group (Fig. 4A–H). Only serum 25(OH)D_3_ and 25(OH)D levels were significantly (p < 0.05) lowered in T2DM group as compared to control (Fig. 4B and G). Results from the present study indicates that T2DM_CAD group patients have more severe vitamin D deficiency as reflected by reduction of all vitamin D metabolites levels in blood compared to T2DM group patients.

### Vitamin D metabolites levels in control, CAD and T2DM_CAD

**Figure 5:**
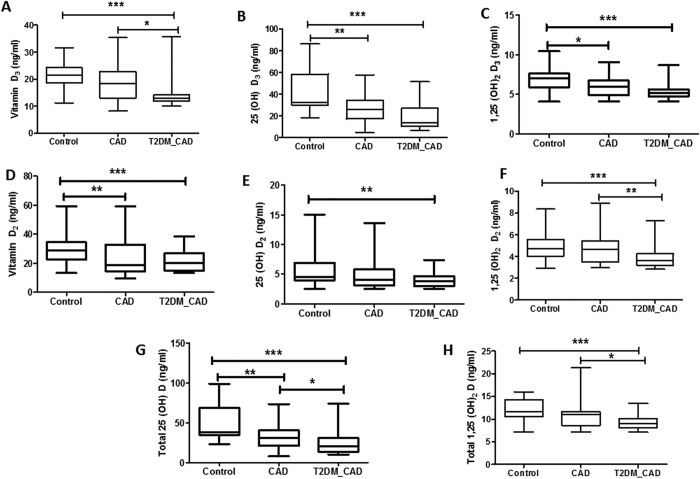
Serum vitamin D metabolites levels in control, CAD and T2DM_CAD groups. Serum levels of vitamin D_3_ (**A**), 25(OH)D_3_ (**B**), 1,25(OH)_2_D_3_ (**C**), vitamin D_2_ (**D**), 25(OH)D_2_ (**E**), 1,25(OH)_2_D_2_ (**F**), 25(OH)D (**G**), and 1,25(OH)_2_D (**H**) were determined in control, CAD, and T2DM_CAD groups. Data were expressed as median and interquartile range. p < 0.05 was considered significant.

All six vitamin D metabolites and total 25(OH)D, total 1,25(OH)_2_D levels in control, CAD and T2DM_CAD group subjects were shown in (Fig. 5A–H). All six Vitamin D metabolites and total 25(OH)D, total 1,25(OH)_2_D were significantly (p < 0.05) lowered in T2DM_CAD subjects when compared with control subjects (Fig. 5A–H). 25(OH)D_3_, 1,25(OH)_2_D_3_,vitamin D_2_, and 25(OH)D metabolites were significantly (p < 0.05) lowered in CAD patients as compared with control subjects (Fig. 5B,C,D and G, respectively). Vitamin D_3_, 1,25(OH)_2_D_2_, 25(OH)D, and 1,25(OH)_2_D metabolites were significantly (p < 0.05) lowered in T2DM_CAD subjects as compared to CAD group subjects (Fig. 5A,F,G and H). These results indicated that T2DM_CAD subjects have severe vitamin deficiency as reflected by reduction of all vitamin D metabolites levels in blood compared with the control subjects.

### Association between 25(OH)D, FBS and HbA1c in all the subjects

**Figure 6:**
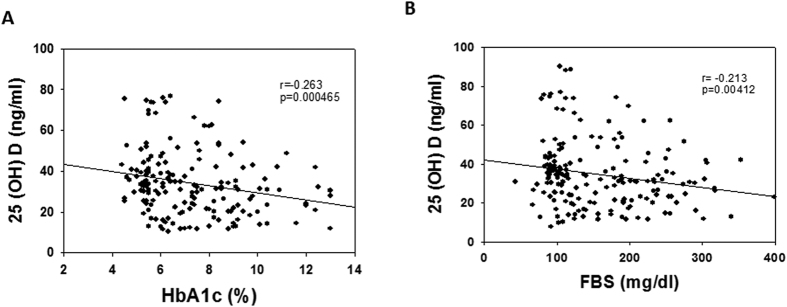
Scatterplot representing relationship between (**A**) 25(OH)D and FBS (**B**) 25(OH)D and HbA1C.

Correlation has been measured to show the association between pairs of variables associated. Spearman correlation is examined between 25(OH)D and HbA1c or FBS. Both pairs of variables showed significant negative correlation between 25(OH)D and FBS (r = −0.213; p < 0.001) (Fig. 6A), and 25(OH)D and HbA1c (r = −0.263; p < 0.001) (Fig. 6B). Present study did not show significant association of 25(OH)D with age, BMI, and CK-MB.

### Correlation Analysis

Supplementary File 1: Table S2 shows vitamin D3 is negatively correlated with creatinine (r = 0.201; p = 0.0063) and positively correlated with eGFR (r = 0.146; p = 0.0480), and gender (r = 0.182; p = 0.0135). 25(OH)D3 is negatively associated with HbA1c (r = −0.250; p = 0.0006), and fasting blood glucose (r = −0.216; p = 0.0033). 1,25(OH)_2_D_3_ is negatively correlated with HbA1c (r = −0.162; p = 0.0283), and fasting blood sugar (r = −0.148; p = 0.0448). Vitamin D_2_ is negatively correlated with gender (r = −0.173; p = 0.0189) and CK-MB (r = −0.161; p = 0.0287). Total 25(OH)D negatively associated with HbA1c (r = −0.263; p = 0.0004), fasting blood glucose (r = −0.213; p = 0.0041) and eGFR (r = −0.339; p = 0.0258) whereas positively correlated with diastolic blood pressure (r = 0.318; p = 0.0258) and creatinine (r = 0.316; p = 0.0267).

### Logistic Regression Analysis

Univariate logistic regression analysis identified several significant factors that predicted the presence of type 2 diabetes with coronary artery diseases. Multivariate logistic regression analysis showed in Table 2, 3, 4, 5. With control and T2DM, multivariate analysis showed HbA1c (odds ratio (OR) 53.01, 95% confidence interval (CI) 47.6–589.2; p < 0.0001), fasting blood glucose (FBS) (OR 1.07, 95% CI 1.03–1.13; p < 0.0001) and total 25(OH)D (OR 0.92, 95% CI 0.85–0.99; p = 0.021) were predictor for T2DM. In addition, we performed force entry multivariate logistic regression analysis to adjust for the factors suspected of contributing to decrease vitamin D metabolites. In the model 1, HbA1c (OR 1000, 95% CI 1.225->999.0; p < 0.0001), FBS (OR 1.350, 95% CI 0.975–1.870; p < 0.0001), smoking history (OR 0.0003, 95% CI 0.0001–16.09; p = 0.0124) and decreased total 25(OH)D (OR 0.694, 95% CI 0.462–1.041; p < 0.0208) were predictors of T2DM. In the model 2, after adjustment for other established risk factors: age, female, smoking history, alcoholic history and family history, showed that HbA1c (OR 120.69 95% CI 2.39->999.99; p = <0.0001), FBS (OR 1.168, 95% CI 1.017–1.342; p < 0.0001) and decreased total 25(OH)D levels (OR 0.817, 95% CI 0.673–0.993; p = 0.0208) were predictors of T2DM (Table 2).

With control and T2DM_CAD, multivariate analysis showed HbA1c (odds ratio (OR) 1000, 95% confidence interval (CI) 0.005–1000; p < 0.0001), fasting blood sugar (OR 1.25, 95% CI 0.89–1.74; p < 0.0001) and total 1,25(OH)_2_D (OR 6.04, 95% CI 0.011–19.20; p = 0.0017) were predictor for T2DM_CAD. In addition, we performed force entry multivariate logistic regression analysis to adjust for the factors suspected of contributing to decrease vitamin D metabolites. In the model 1, creatinine (OR 259.85, 95% CI 7.48–1000; p < 0.0021) and decreased total 25(OH)D (OR 0.951, 95% CI 0.359–0.912; p = 0.019) were predictors of T2DM_CAD. In the model 2, after adjustment for other established risk factors: age, female, smoking history, alcoholic history and family history, showed that age (OR 1.114 95% CI 1.024–1.212; p = 0.0118) and total 1,25(OH)_2_D (OR 0.460, 95% CI 0.242–0.874; p = 0.0177) were predictors of T2DM_CAD (Table 3).

With CAD and T2DM_CAD, multivariate analysis showed HbA1c (odds ratio (OR) 1000, 95% confidence interval (CI) 0.016–1000; p < 0.0001), FBS (OR 1.56, 95% CI 0.960–1.39; p = 0.0061) and total 25(OH)D (OR 0.701, 95% CI 0.42–1.17; p = 0.0411) were predictor for T2DM_CAD. In addition, we performed force entry multivariate logistic regression analysis to adjust the factors suspected of contributing to the decrease of vitamin D metabolites. In the model 1, decreased 1,25(OH)_2_D (OR 0.64, 95% CI 0.44–0.94; p = 0.022) is predictors of T2DM_CAD. In the model 2, after adjustment for other established risk factors: age, female, smoking history, alcoholic history and family history, showed that alcoholic history (OR 10.23 95% CI 1.24–84.47; p = 0.031) is predictors of T2DM_CAD (Table 4).

With T2DM and T2DM_CAD, multivariate analysis showed uric acid (odds ratio (OR) 1.93, 95% confidence interval (CI) 1.03–3.62; p = 0.0413), alcoholic history (OR 48.48, 95% CI 4.89–479.83; p = 0.0009), CK-MB (OR 1.11, 95% CI 1.004–1.24; p = 0.0411), and total 1,25(OH)_2_D (OR 0.314, 95% CI 0.15–0.68; p = 0.0030) were predictor for T2DM_CAD. In addition, we performed force entry multivariate logistic regression analysis to adjust for the factors suspected of contributing to decrease vitamin D metabolites. In the model 1, HbA1C (OR 1.43, 95% CI 1.01–2.02; p = 0.0433), uric acid (OR 2.27, 95% CI 1.25–4.13; p = 0.0069), decreased total 1,25(OH)_2_D (OR 0.53, 95% CI 0.32–0.88; p = 0.0130) were predictors of T2DM_CAD. In the model 2, after adjustment for other established risk factors: age, female, smoking history, alcoholic history, and family history, showed that uric acid (OR 2.82 95% CI 1.05–7.55; p = 0.0392), alcoholic history (OR 26.44, 95% CI 2.12–329.73; p = 0.011), and total 1,25(OH)_2_D (OR 0.29, 95% CI 0.122–0.67; p = 0.0041) were predictors of T2DM_CAD (Table 5).

## Discussion

In the present study, both vitamin D_2_ and vitamin D_3_ metabolites levels were measured in Indian subjects associated with type 2 diabetes and coronary artery diseases. As per the previous literature, in Indian subjects prevalence of vitamin D deficiency is 70–100%, despite living in the 8.4° to 37.6° north latitude and sufficient sunlight exposure throughout the year in most of the places^9^,^10^,^11^,^12^,^13^,^14^,^15^,^16^,^17^. Unlike previous studies, our study found that 83% of control subjects were vitamin D sufficient i.e., more than 30 ng/ml 25(OH)D levels and only 13% subjects were insufficient i.e., below <20 ng/ml. Maximum percentage of healthy subjects have normal range (30–40 ng/ml) of 25(OH)D i.e., within optimum levels. Our results differ from the other Indian studies where maximum number of people were vitamin D deficient^7^,^8^,^9^,^10^,^11^,^12^,^13^,^14^,^15^,^16^,^17^. This difference might be due to the use of different kind of detection methods. Previously, radio immune assay (RIA) method was used to find out vitamin D levels from Indian subjects. However, in the present study UPLC/APCI/HRMS method has been used to measure vitamin D metabolites in Indian subjects.

Type 2 diabetes and coronary artery diseases are two pathological conditions closely related to each other^21^,^22^. Severity of the disease complications generally increases when diabetes coexists with coronary artery diseases. Different type of pathological changes like oxidative stress, advanced end glycation products, hyperglycemia and inflammation are involved in cardiovascular complications in diabetes^23^. Researchers reported that vitamin D deficiency is associated with insulin resistance in type 2 diabetic patients^6^,^24^ and coronary artery disease^25^. Roy *et al*. reported that severe vitamin D deficiency is a risk for acute myocardial infraction in Indians^14^. Recently, Oh *et al*. showed that deletion of vitamin D receptor in macrophage promotes insulin resistance and monocyte cholesterol transport to accelerate atherosclerosis in mice. This study suggests that vitamin D plays important role in inflammation that causes development of atherosclerosis in type 2 diabetes^26^. Herrmann *et al*. reported that serum 25(OH)D is a predictor of macrovascular and microvascular complications in patients with type 2 diabetes^27^. Several other researchers reported that low levels of vitamin D(25(OH)D_3_) were associated with asymptomatic CAD in high-risk type 2 diabetic patients with elevated urinary albumin excretion rate^28^. However, there is no study yet to find the association of vitamin D deficiency with coronary artery diseases in type 2 diabetes. This is the first cross sectional study to compare six vitamin D metabolites and total 25(OH)D, total 1,25(OH)_2_D in control, T2DM, CAD and T2DM with CAD patients from south part of India.

The present study shows that 83% and 73% of control subjects have sufficient total 25(OH)D (>30 ng/ml) and total 1,25(OH)_2_D (>11 ng/ml) levels, respectively. However, the percentage of diabetes and diabetes with CAD subjects were increased when we considered insufficient 25(OH)D and 1,25(OH)D_2_ levels. All the vitamin D metabolites were significantly lowered in T2DM with CAD subjects when compared with control and T2DM subjects. However, only 25(OH)D_3_ and total 25(OH)D levels were significantly decreased in the T2DM subjects as compared with control subjects. The study also indicated that T2DM patients with total 1,25(OH)_2_D deficiency are more prone to coronary artery diseases. It was observed that deficiency of vitamin D metabolites were associated with coronary artery diseases among diabetic patients. However, this deficiency is much less for T2DM or CAD alone. Significant negative correlation was observed between total 25(OH)D and HbA1c or FBS but not with BMI. Similar to our study, other studies also showed that total 25(OH)D levels were negatively correlated with HbA1C and blood glucose^29^ but not with the BMI^30^. Interestingly, our data found a significant correlation between vitamin D metabolites and the prevalence of CAD among T2DM patients.

To further confirm the causal relationship between lower levels of plasma vitamin D metabolites and T2DM or T2DM with CAD, multivariate logistic regression was performed. The analysis of our results revealed that while lower serum 25(OH)D predicts type 2 diabetes, lower serum 1,25(OH)_2_D predicts coronary artery diseases in type 2 diabetes. Although, we have not able to investigate the mechanism behind the role of vitamin D on diabetes and CAD, several previous literatures shed light regarding the molecular effect of Vitamin D. 1,25(OH)2D stimulates the expression of insulin receptors, which in turn will affect insulin sensitivity^31^. 1,25(OH)2D may also enhance insulin sensitivity by activating peroxisome proliferator-activated receptor delta (PPAR-δ), which is a transcription factor regulates the metabolism of fatty acids in skeletal muscle and adipose tissue^32^. Vitamin D insufficiency has been associated with increased fat infiltration in skeletal muscle and contributes to decrease in insulin sensitivity^33^. Similar to diabetes, lower concentration of 1,25(OH)2D is associated with athreosclerosis and coronary artery disease through affecting vitamin D receptor signaling. Impairment of VDR signaling accelerates foam cell formation in diabetics^26^.

Unlike previous studies, the strength of the present study is to measure six vitamin D metabolites by novel and specific mass spectrometry assay in four different groups like control, T2DM, CAD, and T2DM with CAD.

## Limitations

Several limitations of our study should be noted. (1) It was a single-center one-point time study from South part of India. (2) We have small sample size in each group. (3) Although we adjusted for potential confounders, we cannot eliminate the possibility of residual confounding factors influencing our results. (4) We have not measured parathyroid hormone and fibroblast growth factor (FGF-23) levels in blood. (5) This study is an observational study and does not address causality.

## Conclusion

In conclusion, vitamin D metabolites were decreased in T2DM with CAD subjects as compared with control, T2DM, and CAD subjects. Vitamin D metabolites like 25(OH)D can be used to predict T2DM while 1,25(OH)_2_D can be used to predict CAD in T2DM. Further studies are required to show that vitamin D supplementation may be beneficial to reduce coronary artery diseases among diabetic patients.

## Materials and Methods

### Patient Selection

In the present cross sectional study, serum samples from 187 patients, including male and female aged 35–65 years, were randomly taken from the Mediciti Hospital, Hyderabad, India.

Subjects were allocated into four study groups as mentioned below:

**Group 1:** Control (n = 50).

**Group 2:** Type 2 diabetes mellitus (T2DM, n = 71).

**Group 3:** Coronary artery disease (CAD, n = 28).

**Group 4:** Coronary artery disease with Type 2 diabetes mellitus (T2DM_CAD, n = 38).

### Inclusion and Exclusion Criteria

**Group 1** Control subjects had no prior history of T2DM, hypertension, coronary artery diseases or any other cardiovascular diseases, and were not taking medication for any chronic medical condition. Fasting blood glucose, HbA1c and blood chemistry were normal. **Group 2** (T2DM) included subjects with HbA1c levels ≥ 6.5% as per American Diabetes Association (ADA) guidelines with proven history of T2DM but no other complications. **Group 3** (CAD) included subjects with narrowing or blockage of one or more epicardial coronary artery with greater than 25% stenosis shown in coronary angiography and diagnosed by cardiologists^3^. This group had no prior history of T2DM. **Group 4** (T2DM_CAD) included subjects with coronary artery disease as defined for group 3 but patient had HbA1c levels ≥6.5% and prior history of T2DM^3^. Exclusion criteria defined for this study were clinical or laboratory evidence of liver failure, renal failure (plasma creatinine levels >1.5 mg/dl), type 1 diabetes, cancer, thyroid disease and pregnancy; and subjects on vitamin D treatment therapy were also excluded in this study. The study conforms to the principles outlined in the Declaration of Helsinki and was approved by the Mediciti Ethics Committee (Institutional Ethics Committee), Hyderabad. All patients were given detailed information of the study. Informed consent was obtained from all the subjects.

### Selection process for patients with coronary artery diseases

Coronary artery disease patients were identified in the cardiac catheterization unit of Mediciti Hospital. After coronary angiogram, all patients were evaluated by cardiologists in the inpatient setting. If patients were found to have any evidence of CAD, demographic, clinical, and angiographic data were collected from all such patients. Fasting samples were collected prior to the percutaneous coronary intervention or coronary artery bypass graft.

### Sample collection process

Clinical history and complete physical examination including measurements of blood pressure were collected and conducted for all the participants. Blood samples were collected by venipuncture after an overnight fast, using Becton Dickison Vacutainer^®^ Red colour coded tubes and blood was allowed to clot by leaving at room temperature for 15 min. After centrifugation at 1500 g for 15 min at 4 °C, serum was collected in 1.2 ml cryo vials and immediately stored at −80 °C until testing.

### Clinical and biochemical parameters

Height and weight were obtained using standardised techniques and instruments. The body mass index (BMI) was calculated as the weight in kilograms divided by the square of the height in meters [weight (kg)/Height (m^2^)]. Fasting blood glucose levels were measured by the Free Style optimum glucometer (Abbott Diabetes Care, Australia). HbA1c levels were measured by the Bayer A1C Now^+^ Multi test A1C system (Catalog no.08842610). Creatinine, uric acid, total cholesterol, triglycerides, high density lipoprotein (HDL) cholesterol, and CK-MB levels were measured by Siemens automated analyser (Dimension Xpand^plus^). Estimated glomerular filtration rate (eGFR) calculated from creatinine by using Modification of Diet in Renal Disease (MDRD) formula. Low density lipoprotein (LDL) cholesterol concentrations were calculated by using the Friedewald formula. ApoB (mg/dl) was calculated based on the formula ApoB  =  −33.12  +  0.675*LDL  +  11.95*ln (Triglycerides)^34^.

### Sample preparation and analytical method for estimation of vitamin D metabolites

Serum vitamin D metabolites concentrations were determined by liquid chromatography–Orbitrap mass spectrometry (UPLC/APCI/HRMS). Stock solutions of vitamin D_3_ and vitamin D_2_ and its respective metabolites (25(OH)D_3_, 1,25(OH)_2_D_3_, 25(OH)D_2_ and 1,25(OH)_2_D_2_) were prepared in ethanol; Calibration curve (5–200 ng/ml) and quality control sample was prepared from stock solution using methanol; Similarly the dihydrotachysterol 50 ng/ml was used as an internal standard (IS).

100 μl of serum (test sample) was added with 10 ul of IS solution followed by 1 ml of hexane:heptane:acetone in the ratio of 45:40:15. After vertex it properly, the solution was incubated in a shaker for 10 min. The supernatant was separated after centrifugation at 6000 rpm at 4 °C. Supernatant was collected and evaporated using scan vac speed vacuum concentrator. After re-dissolving the concentrate sample in 100 μl of methanol, 10 ul of dissolved sample was used for analysis.

Separation of metabolites was achieved using waters Xselect CSH phenyl hexyl column (150 mm*4.6 mm ID; particle size 3.5 um) with gradient mode. Both mobile phase i.e., mobile phase A (10 mM of ammonium formate in methanol) and mobile phase B (ACN: Acetone: IPA (5:4:1)) were set as follows: (T_min_/% proportion of solvent (B): _0_/5, _0–5_/95, _5–6_/5, and _6–8_/5. The flow rate of the mobile phase was 1.00 ml/min while the column temperature and the injection volume was set as 25 °C and 10 μL, respectively. Mass spectrometric detection was carried out by Orbitrap mass analyzer (Exactive Thermo Scientific, Germany) equipped with an atmospheric pressure chemical ionization (APCI) source. The data acquisition was under the control of Xcalibur software. The typical operating source conditions for MS scan in positive ion APCI mode were optimized as follows sheath gas flow rate 65; Aux gas flow rate 20; Discharge current 10.00 μA; Capillary temperature 300 °C; Capillary voltage 50 V; Tube lens voltage 85.0 V; Skimmer voltage 18.00 V and vaporizer temperature 380 °C. For full scan MS mode, the mass range was set at *m/z* 100–500. All the spectra were recorded under identical experimental conditions and scan rate of 4.9 scan/sec. The full-scan mode across *m/z* 250–500 that include vitamin D, its metabolites and IS.

For quantification, EICs of [M + H]^+^ at *m/z* 385.34649, [M + H − H_2_O]^+^ at *m*/*z* 383.33084 and [M + H − H_2_O]^+^ at *m*/*z* 399.32576 for VitD_3_, 25(OH)D_3_ and 1,25(OH)D_3,_ respectively with a 5 ppm range centered on the exact *m*/*z* value were generated. Similarly, EICs of [M + H − H_2_O]^+^ at *m*/*z* 379.33593, [M + H − H_2_O]^+^ at *m*/*z* 395.33084 and [M + H – H_2_O]^+^ at *m*/*z* 411.32576, and [M + H − H_2_O]^+^ at *m*/*z* 381.31519 for VitD_2_, 25(OH)D_2_ and 1,25(OH)D_2_ and IS, respectively (Supplemental File: Figs S1–S7). The developed method was validated according to U.S Food and Drug Administration (FDA) guideline for the validation of the bioanalytical method. Details of the method were provided briefly in supplementary file (Supplemental File 1: Table S1).

### Statistical analysis

Normally distributed variables were summarized as mean and standard deviation, while non-normally distributed variables were expressed as median and interquartile ranges. One-way analysis of variance (ANOVA) was used to determine whether there are any significant differences between the means of three independent (unrelated) groups of 25(OH)D. Anthropometric and clinical characteristics were presented by 25(OH)D categories (>30, 20–30 and <20 ng/ml). Kruskal-Wallis test was used for comparing non-normally distributed variables with post-hoc Dunn’s multiple comparison test. One-way ANOVA with Bonferroni test was used for normally distributed variable. Kruskal-Wallis test was used to compare vitamin D metabolites levels in control, CAD and T2DM_CAD groups and control, T2DM and T2DM_CAD groups. Spearman rank order correlation was used to find correlation among 25(OH)D, HbA1c, FBS and BMI. Total 25(OH)D [sum of 25(OH)D+ 25(OH)D_3_] and total 1,25(OH)_2_D [sum of 1,25(OH)_2_D_2_ + 1,25(OH)_2_D_3_] were evaluated for overall comparison. Multivariate logistic regression analysis was also performed to test the association of the serum 25(OH)D level with the presence of T2DM (i.e. the dependent variable). Two forced-entry multivariate regression models were performed (i.e. unadjusted and adjusted). The dependent variable was involved as a compound end point: patients with T2DM or CAD or both complications (coded as 1) and patients with neither T2DM nor CAD (coded as 0 i.e. control). Univariate analysis was used to identify covariates as potential confounders on the basis of their significance (p < 0.05). ORs with 95% CIs were used to report the results. Distribution data were presented with Origin 6.0. Box and whisker plots were made with GraphPad Prism version 5.01. Statistical analysis was performed with SAS^®^ V.9.4 software.

## Additional Information

**How to cite this article**: Adela, R. *et al*. Lower Vitamin D Metabolites Levels Were Associated With Increased Coronary Artery Diseases in Type 2 Diabetes Patients in India. *Sci. Rep.* **6**, 37593; doi: 10.1038/srep37593 (2016).

**Publisher’s note:** Springer Nature remains neutral with regard to jurisdictional claims in published maps and institutional affiliations.

## Supplementary Material

Supplementary Information

## Figures and Tables

**Figure 1:**
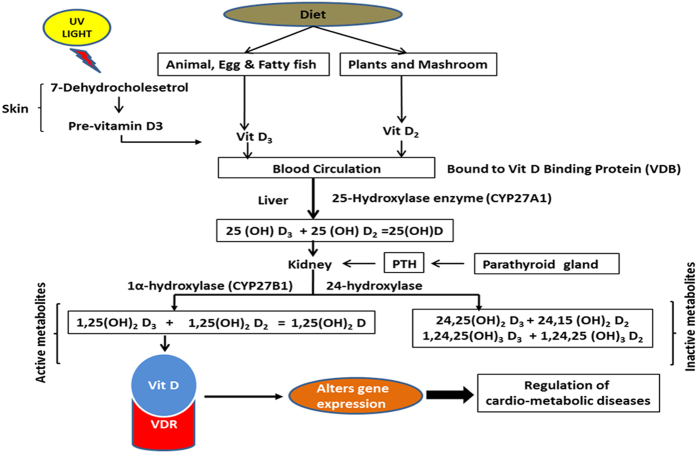
Vitamin D synthesis and metabolism: Vitamin D_2_ is synthesized from food whereas D_3_ is synthesized from 7-dehydrocholesterol by sun light. Vitamin D_2_ and D_3_ hydroxylate at 25 position in the liver to form 25(OH)D_2_ and 25(OH)D_3_, then hydroxylate again at position 1α to form active metabolites 1,25(OH)_2_D_2_ and 1,25(OH)_2_D_3_. These active metabolites participate in the biological activity.

**Table 1:** Anthropometric and biochemical variables by serum 25(OH)D concentrations (>30 and 20–30 and <20 ng/ml).

Variables	All measured participants	25(OH)D ng/ml			p value
		>30 ng/ml	20–30 ng/ml	<20 ng/ml	
N (%)	187	111 (59.4)	40 (21.4)	36 (19.3)	—
Age (years)	47.8 (8.9)	47.4 (9.6)	48.1 (8.4)	48.8 (7.0)	0.696
Gender (Male/Female)	126/61	74/37	25/50	27/9	—
BMI	26.4 (4.6)	26.3 (4.7)	26.6 (5.0)	26.4 (4.0)	0.939
Systolic BP (mmHg)	132.5 (19.2)	131.9 (16.9)	135.4 (24.7)	131.2 (18.9)	0.565
Diastolic BP (mmHg)	81.8 (9.8)	81.8 (9.8)	82.2 (10.7)	81.7 (9.3)	0.972
HbA1C (%)	6.5 (5.5–8.5)	6.2 (5.4–8.1)	7.4 (5.3–88)	6.9 (5.6–8.9)	0.0403^*^
FBS (mg/dl)	130 (99–201)	113 (97–190)	174.5 (109.5–235)	138.5 (105–200)	0.0622
Creatinine (mg/dl)	0.9 (0.7–1)	0.9 (0.7–1)	0.85 (0.8–0.95)	0.9 (0.75–1.2)	0.114
eGFR (mL/min/1.73 m2)	92.9 (25.1)	94.4 (25.6)	94.8 (20.1)	86.7 (28.2)	0.245
Uric acid (mg/dl)	4.6 (1.5)	4.8 (1.5)	4.3 (1.5)	4.4 (1.3)	0.159
TG (mg/dl)	152 (108–226)	160 (113–237)	136.5 (103.5–172)	177 (109.5–242.5)	0.137
HDL-C (mg/dl)	33 (28–39)	33 (29–39)	36.5 (30–42.5)	31.5 (26–36)	0.142
CK-MB (mg/dl)	21.5 (8.5)	21.2 (7.4)	21.9 (8.6)	21.9 (10.1)	0.869
Apo-B (mg/dl)	81.7 (68.3–96.6)	84.7 (71.8–99.6)	77 (64.2–92.2)	68.4 (65.7–88.7)	0.173
25(OH)D (ng/ml)	36 (19.5)	46.5 (18)	24.5 (3.1)	14.1 (2.9)	<0.001^*^
1,25(OH)_2_D (ng/ml)	11.1 (9.2–12.3)	11.6 (10.1–14.2)	10.6 (8.9–11.6)	8.3 (8–9.7)	<0.001^*^
Vitamin D_2_ (ng/ml)	27.4 (10.5)	29.7 (10.3)	26 (10.9)	20.9 (7)	<0.001^*^
25(OH)D_2_ (ng/ml)	4.5 (3.4–5.9)	4.5 (3.4–7.3)	4.5 (3.6–5.9)	3.5 (2.9–4.5)	0.0002^*^
1,25(OH)_2_D_2_ (ng/ml)	4.4 (3.7–5.4)	4.8 (4.0–6.1)	4.2 (3.9–4.8)	3.4 (3.1–4.0)	<0.001^*^
Vitamin D_3_ (ng/ml)	18.9 (31.8–23.3)	22 (18.3–24.5)	16.3 (13.1–19.2)	13.3 (12–15)	<0.001^*^
25 (OH)D_3_ (ng/ml)	27.4 (18.3–37.5)	33.4 (28.5–45–4)	19.4 (17.4–22)	10.5 (8.3–11.9)	<0.001^*^
1,25(OH)_2_D_3_ (ng/ml)	6.3 (5.1–7.3)	6.8 (5.7–7.5)	5.6 (4.9–7.1)	5.1 (4.7–5.3)	<0.001^*^

Data are Mean (SD) and Median (Q1–Q3) for normally distributed & non-normally distributed variables, respectively. *p < 0.05 for a test comparing across 25(OH)D groups. Significant values were shown in Bold.

**Table 2:** Logistic regression analysis of T2DM vs control subjects.

Factor	Univariate		Multivariate		Model 1 (unadjusted)		Model 2 (adjusted)	
	OR (95% CI)	P value	OR (95% CI)	P value	OR (95% CI)	P value	OR (95% CI)	P value
Age	1.03 (0.99–1.08)	0.1586			1.29 (0.826–2.029)	0.0955		
Sex (female)	1.83 (0.87–3.84)	0.1107			0.001 (0.0001–6.200)	0.0600		
BMI	1.06 (0.99–1.15)	0.1165						
HbA1c (%)	15.49 (5.37–44.69)	<0.0001	**53.01 (47.69–589.25)**	**<0.0001**	**1000 (1.23->999.99)**	**<0.0001**	**120.69 (2.39->999.99)**	**<0.0001**
FBS (mg/dl)	1.09 (1.05–1.13)	<0.0001	**1.07 (1.03–1.13)**	**<0.0001**	**1.350 (0.98–1.87)**	**<0.0001**	**1.17 (1.02–1.34)**	**<0.0001**
Systolic BP (mmHg)	1.05 (1.02–1.08)	0.0002			1.34 (0.98–1.83)	0.1699	1.18 (0.98–1.40)	0.1699
Diastolic BP (mmHg)	1.07 (1.02–1.12)	0.0024						
Creatinine (mg/dl)	1.77 (0.46–6.88)	0.41						
eGFR (ml/min/1.72 m2)	0.98 (0.97–1.00)	0.121						
Uric acid (mg/dl)	0.76 (0.58–0.99)	0.0451						
CK-MB (u/l)	0.93 (0.88–0.98)	0.0101			0.32 (0.09–1.18)	0.1364	0.666 (0.415–1.068)	0.1364
Apo-B (mg/dl)	0.99 (0.99–1.005)	0.5924						
Smoking history (years)	0.86 (0.31–2.36)	0.7708			**0.003 (0.0001–16.09)**	**0.0124**		
Alcoholic history (years)	0.78 (0.33–1.86)	0.5728						
Family history (years)	1.46 (0.69–3.07)	0.319						
D3 (ng/ml)	0.99 (0.93–1.06)	0.9288						
25(OH)D3 (ng/ml)	0.97 (0.94–0.99)	0.0022						
1,25(OH)D3 (ng/ml)	0.90 (0.73–1.11)	0.3365						
D2 (ng/ml)	1.01 (0.98–1.05)	0.5280						
25(OH)D2 (ng/ml)	0.92 (0.82–1.03)	0.1555						
1,25(OH)D2 (ng/ml)	1.04 (0.85–1.28)	0.6840						
Total 25(OH)D (ng/ml)	0.97 (0.95–099)	0.0019	**0.92 (0.85–0.99)**	**0.021**	**0.69 (0.46–1.04)**	**0.0208**	**0.82 (0.68–0.99)**	**0.0208**
Total 1,25(OH)D (ng/ml)	0.98 (0.87–1.71)	0.7390						

**Model 1:** unadjusted, **Model 2:** adjusted for age, sex, smoking history, alcoholic history and family history of diabetes.

**Table 3:** Logistic regression analysis of T2DM_CAD vs control subjects.

Factor	Univariate		Multivariate		Model 1 (Unadjusted)		Model 2 (Adjusted)	
	OR (95% CI)	P value	OR (95% CI)	P value	OR (95% CI)	P value	OR (95% CI)	P value
Age	1.01 (1.04–1.15)	0.001					**1.114 (1.024–1.212)**	**0.0118**
Sex (female)	0.27 (0.9–0.81)	0.019					0.326 (0.040–2.666)	0.2960
BMI	0.98 (0.88–1.073)	0.602						
HbA1c (%)	23.31 (3.91–138.99)	0.0005	**1000 (0.005–1000)**	**<0.0001**				
FBS (mg/dl)	1.07 (1.04–1.10)	<0.0001	**1.25 (0.89–1.74)**	**0.0001**				
Systolic BP (mmHg)	1.04 (1.01–1.07)	0.023						
Diastolic BP (mmHg)	1.06 (0.99–1.12)	0.067						
Creatinine (mg/dl)	20.49 (2.42–173.36)	0.0056			**259.85 (7.48–1000)**	**0.0021**	32.932 (0.459–1000)	0.1089
eGFR (ml/min/1.72 m2)	0.98 (0.95–0.99)	0.012						
Uric acid (mg/dl)	0.90 (0.67–1.22)	0.493						
CK-MB (u/l)	5.83 (2.17–15.68)	0.0005					9.903 (0.714–137.319)	0.0875
Apo-B (mg/dl)	6.09 (2.39–15.48)	0.0001					0.755 (0.072–8.340)	0.8335
Smoking history (years)	1.16 (0.49–2.77)	0.739					1.281 (0.255–6.440)	0.7636
Alcoholic history (years)	1.02 (0.97–1.08)	0.349						
Family history (years)	0.95 (0.92–0.98)	0.0003						
D3 (ng/ml)	0.94 (0.88–1.003)	0.059						
25(OH)D3 (ng/ml)	0.88 (0.83–0.93)	<0.0001						
1,25(OH)D3 (ng/ml)	0.354 (0.22–0.56)	<0.0001						
D2 (ng/ml)	0.88 (0.82–0.94)	<0.002						
25(OH)D2 (ng/ml)	0.92 (0.80–1.05)	0.211						
1,25(OH)D2 (ng/ml)	0.39 (0.23–0.67)	0.0006						
Total 25(OH)D (ng/ml)	0.91 (0.86–0.95)	<0.0001			0.951 (0.899–1.005)	0.074	0.960 (0.902–1.022)	0.1986
Total 1,25(OH)D (ng/ml)	0.503 (0.37–0.68)	<0.0001	**6.04 (0.011–19.20)**	**0.0017**	**0.572 (0.359–0.912)**	**0.019**	**0.460 (0.242–0.874)**	**0.0177**

**Model 1:** unadjusted, **Model 2:** adjusted for age, sex, smoking history, alcoholic history and family history of diabetes.

**Table 4:** Logistic regression analysis of T2DM_CAD vs CAD subjects.

Factor	Univariate		Multivariate		Model 1 (unadjusted)		Model 2 (adjusted)	
	OR (95% CI)	P value	OR (95% CI)	P value	OR (95% CI)	P value	OR (95% CI)	P value
Age	1.07 (0.99–1.14)	0.0541					1.08 (0.99–1.16)	0.072
Sex (female)	2.05 (0.37–11.38)	0.4142					8.69 (0.38–197.01)	0.175
BMI	1.11 (0.97–1.27)	0.1396						
HbA1c (%)	243.56 (6.08- >999)	0.0035	**1000 (0.02–1000)**	**<0.0001**				
FBS (mg/dl)	1.06 (1.03–1.09)	0.0002	**1.16 (0.96–1.39)**	**0.0061**				
Systolic BP (mmHg)	1.00 (0.98–1.03)	0.8368						
Diastolic BP (mmHg)	0.99 (0.95–1.05)	0.9306						
Creatinine (mg/dl)	0.89 (0.33–2.45)	0.8318						
eGFR (ml/min/1.72 m2)	0.99 (0.97–1.02)	0.9117						
Uric acid (mg/dl)	0.68 (0.48–0.96)	0.0276						
CK-MB (u/l)	1.01 (0.95–1.06)	0.8099						
Apo-B (mg/dl)	0.99 (0.97–0.02)	0.6418						
Smoking history (years)	0.89 (0.33–2.39)	0.8158					0.52 (0.07–3.77)	0.518
Alcoholic history (years)	2.40 (0.87–6.62)	0.0896					**10.23 (1.24–84.47)**	**0.031**
Family history (years)	2.87 (0.89–9.23)	0.0771					3.96 (0.91–17.29)	0.067
D3 (ng/ml)	0.98 (0.94–1.03)	0.5086						
25(OH)D3 (ng/ml)	0.96 (0.92–0.99)	0.0284						
1,25(OH)D3 (ng/ml)	0.59 (0.37–0.93)	0.0220						
D2 (ng/ml)	0.99 (0.94–1.04)	0.6456						
25(OH)D2 (ng/ml)	0.96 (0.87–1.06)	0.4450						
1,25(OH)D2 (ng/ml)	0.50 (0.31–0.82)	0.0063						
Total 25(OH)D (ng/ml)	0.96 (0.93–0.99)	0.0346	**0.70 (0.42–1.17)**	**0.0411**	0.99 (0.96–1.05)	0.96	0.98 (0.93–1.05)	0.652
Total 1,25(OH)D(ng/ml)	0.64 (0.47–0.87)	0.0040			**0.64 (0.44–0.94)**	**0.022**	**0.58 (0.35–0.95)**	**0.031**

**Model 1:** unadjusted, **Model 2:** Adjusted for age, sex, smoking history, alcoholic history and family history of diabetes.

**Table 5:** Logistic regression analysis of T2DM_CAD vs T2DM subjects.

Factor	Univariate		Multivariate		Model 1 (unadjusted)		Model 2 (adjusted)	
	OR (95% CI)	P value	OR (95% CI)	P value	OR (95% CI)	P value	OR (95% CI)	P value
Age	1.07 (1.01–1.14)	0.026					1.09 (0.98–1.21)	0.131
Sex (female)	0.16 (0.05–0.06)	0.0032					0.59 (0.07–4.88)	0.629
BMI	0.92 (0.82–1.03)	0.1411						
HbA1c (%)	1.29 (0.97–1.73)	0.075			**1.43 (1.01–2.02)**	**0.0433**	1.58 (0.92–2.69)	0.095
FBS (mg/dl)	1.002 (0.99–1.01)	0.539						
Systolic BP (mmHg)	1.03 (1.00–1.06)	0.047						
Diastolic BP (mmHg)	1.05 (0.99–1.12)	0.0841						
Creatinine (mg/dl)	12.74 (1.30–124.5)	0.0287						
eGFR (ml/min/1.72 m2)	0.98 (0.96–1.003)	0.103					1.02 (0.98–1.06)	0.301
Uric acid (mg/dl)	1.46 (0.99–2.16)	0.056	**1.93 (1.03–3.62)**	**0.0413**	**2.27 (1.25–4.13)**	**0.0069**	**2.82 (1.05–7.55)**	**0.0392**
CK-MB (u/l)	8.89 (2.28–34.59)	0.0016						
Apo-B (mg/dl)	15.39 (3.89–60.8)	<0.001	**48.48 (4.89–479.83)**	**0.0009**			**26.44 (2.12–329.73)**	**0.011**
Smoking history (years)	0.702 (0.26–1.90)	0.487						
Alcoholic history (years)	1.12 (1.04–1.22)	0.0051	**1.11 (1.004–1.24)**	**0.0411**	**1.13 (1.03–1.24)**	**0.0125**	1.10 (0.98–1.24)	0.1109
Family history (years)	0.98 (0.95–1.01)	0.1734						
D3 (ng/ml)	1.00 (0.94–1.05)	0.994						
25(OH)D3 (ng/ml)	0.98 (0.94–1.02)	0.364						
1,25(OH)D3 (ng/ml)	0.64 (0.39–1.03)	0.068						
D2 (ng/ml)	0.91 (0.84–0.98)	0.0083						
25(OH)D2 (ng/ml)	1.03 (0.91–1.156)	0.6803						
1,25(OH)D2 (ng/ml)	0.58 (0.33–1.01)	0.0547						
Total 25(OH)D (ng/ml)	0.99 (0.95–1.03)	0.5163						
Total 1,25(OH)D (ng/ml)	0.69 (0.48–0.99)	0.0477	**0.314 (0.15–0.68)**	**0.0030**	**0.53 (0.32–0.88)**	**0.0130**	**0.29 (0.122–0.67)**	**0.0041**

**Model 1:** unadjusted, **Model 2**: Adjusted for age, sex, and alcoholic history.
