# Supplementary material for: Lower Vitamin D Metabolites Levels Were Associated With Increased Coronary Artery Diseases in Type 2 Diabetes Patients in India
Source: Sci Rep. 2016 Nov 24;6:37593. doi: 10.1038/srep37593 (PMC5121614; doi:10.1038/srep37593)
Supplement: Supplementary Information [file srep37593-s1.doc]

**Title: Lower vitamin D metabolites levels were associated with increased coronary artery diseases in type 2 diabetes patients in India**

Ramu Adela^1^, Roshan M Borkar^2^, Murali Mohan Bhandi^2^, Gayatri Vishwakarma^3^; P. Naveen Chander Reddy^4^, R. Srinivas^3^*, Sanjay K Banerjee^1^*

^1^Drug Discovery Research Center, Translational Health Science and Technology Institute (THSTI), Faridabad, HR-121001, India (RA, SKB)

^2^National Center for Mass Spectrometry, Indian Institute of Chemical Technology (CSIR-IICT), Hyderabad, India (RMB, MMB, RS).

^3^Clinical Development Service Agency (CDSA), Translational Health Science and Technology Institute (THSTI), Faridabad, HR-122016, India (GV).

^4^Cardiology Division, Mediciti Institute of Medical Sc Hospital, Hyderabad-500063, India (PNCR).

Ramu Adela

[ramuadela@gmail.com](mailto:ramuadela@gmail.com)

Roshan M Borkar

[roshanudps@gmail.com](mailto:roshanudps@gmail.com)

Murali Mohan Bhandi

[bmmreddy34@gmail.com](mailto:bmmreddy34@gmail.com)

Gayatri Vishwakarma

[gayatrivishwakarma.cdsa@thsti.res.in](mailto:gayatrivishwakarma.cdsa@thsti.res.in)

P.Naveen Chander Reddy

[podduturunaveen@yahoo.co.in](mailto:podduturunaveen@yahoo.co.in)

Dr.Sanjay K Banerjee

[skbanerjee@thsti.res.in](mailto:skbanerjee@thsti.res.in)

Dr. R. Srinivas

[sragampeta@yahoo.co.in](mailto:sragampeta@yahoo.co.in)

**Sample Size calculation**

187 subjects were recruited based on consecutive sampling technique including 10% missing data considerations from Mediciti Hospital, Hyderabad India. The sample size produced a margin of error of 7.3% of the study with confidence limit of 95% with on-line sample size calculator [Raosoft Inc. [http://www.raosoft.com/samplesize.html], accessed on 15^th^ August 2014].

**Methods**

**Sample Preparation for Vitamin D Metabolites Measurement**

**Vitamin d stripped serum preparation**

Biocell serum was stripped using a method adapted by the Carter with slight modification. A 15 mL aliquot of Biocell serum was mixed with 0.5 g of activated charcoal (untreated powder, 100–400 mesh) by agitation at room temperature overnight. The serum was then centrifuged at 9000**×**g for 20 min. The supernatant was removed and filtered with a syringe connected to an Acrodisc Syringe (Supor membrane 0.2µM). The filtered syringe was again centrifuged at 6000 RPM and the supernatant was separated. Only 10 µL of triple stripped Biocell serum (pure serum) was run into the UPLC/APCI/HRMS method to confirm undetectable level of vitamin D_3_ (VitD_3_) and vitamin D_2_ (VitD_2_) and its respective metabolites.

**Preparation of stock solutions, calibration standard solutions and control samples**

The stock solution of VitD_3_, VitD_2_ and its respective metabolites (25(OH)D_3_, 1,25(OH)_2_ D_3_ and 25(OH)D_2_ and 1, 25(OH)_2_ D_2_) was prepared in ethanol. Serial dilutions were made, using this stock solution, to prepare the primary aliquots of vitamin D and its metabolites in methanol for calibration curve and quality control (QC) samples. Similarly, stock solution of 1 mg/mL of Dihydrotachysterol (Internal standard, IS) was also prepared in ethanol, further diluted with methanol to prepare working solution containing a concentration of 50 ng/mL.

**Preparation of calibration and quality control (qc) samples**

10 µL of primary aliquot of VitD_3_, VitD_2_, 25(OH)D_3_, 1,25(OH)_2_ D_3_ and 25(OH)D_2_ and 1, 25(OH)_2_ D_2_ were spiked in 80 µL of vitamin D stripped serum to yield calibration curve samples ranging from 3-200 ng/mL for VitD_3_, 25(OH)D_3_ and VitD_2_ and 5-200 ng/mL for 1,25(OH)_2_ D_3_ and 25(OH)D_2_ and 1,25(OH)_2_D_2_. Similarly, low, middle and high QC samples (LQC, MQC and HQC) at three different levels were prepared independently at concentrations of 3 ng/mL (LQC); 10 ng/mL (MQC); 200 ng/mL (HQC) for VitD_3_, 25(OH) D_3_ and VitD_2_. Whereas, 5 ng/mL (LQC); 10 ng/mL (MQC); 200 ng/mL (HQC) for 1,25(OH)_2_ D_3_ and 25(OH)D_2_ and 1, 25(OH)_2_ D_2_. All the stock solutions were stored at 0-4^o^C for further use.

**Extraction of vitamin d and its metabolites from serum**

To an aliquot of 100 µL of serum (calibration standard and test sample), 10 µL of IS solution was added at a concentration of 50 ng/mL followed by 1 mL of hexane: heptane: acetone in the ratio of 45:40:15. This mixture was thoroughly mixed. The mixture solutions were kept in shaker for 10 min followed by centrifugation at 6000 rpm at 4 °C and then supernatant layer were taken into another eppendorf tube. Evaporation of supernatant was done on ScanVac speed Vacuum Concentrator and 100 µL of methanol was added. Only 10 µL aliquots of sample solution were injected into LC/MS system for analysis.

**UPLC/APCI/HRMS conditions**

Analysis was carried out on U-HPLC instrument (Thermo Scientific Accela, Germany) equipped with a quaternary pump, a de-gasser, a diode-array detector, an auto sampler and a column compartment. Mass spectrometric detection was carried out using an Orbitrap mass analyzer (Exactive Thermo Scientific, Germany) equipped with an atmospheric pressure chemical ionization (APCI) source. The data acquisition was under the control of Xcalibur software. The separation of vitamin D and its metabolites and IS from endogenous substances were achieved using Water’s X select CSH phenyl hexyl column (150 mm x 4.6 mm I.D.; particle size 3.5 µm) and mobile phase consisting of a mixture of ammonium formate 10 mM in methanol (A ) and Acetonitrile: Acetone: IPA (5:4:1) (B) in an gradient program mode. The gradient solvent programme was set as follows: (T_min_/ % proportion of solvent B): _0_/5, _0-5_/95, _5-6_/5, and _6-8_/5. The flow rate of the mobile phase was 1.00 ml/min, the column temperature 25°C and the injection volume 10 μL. Compound dependent parameters and instrumental parameters were optimized by infusing neat solutions of individual vitamin D, metabolites and the IS by using a syringe pump.

The typical operating source conditions for MS scan in positive ion APCI mode were optimized as follows sheath gas flow rate 65; Aux gas flow rate 20; Discharge current 10.00 µA; Capillary temperature 300 °C; Capillary voltage 50 V; Tube lens voltage 85.0 V; Skimmer voltage 18.00 V and vaporizer temperature 380 °C. For full scan MS mode, the mass range was set at *m/z* 100-500. All the spectra were recorded under identical experimental conditions and scan rate of 4.9 scan/sec. The full-scan mode across *m/z* 250–500 that include vitamin D, its metabolites and IS.

For quantification, EICs of [M+H] ^+^at *m/z* 385.34649,[M+H-H_2_O]^+^at *m/z* 383.33084 and [M+H-H_2_O]^+^ at *m/z* 399.32576 for VitD_3_, 25(OH) D_3_ and 1,25(OH)_2_ D_3_, respectively with a 5 ppm range centered on the exact *m/z* value were generated. Similarly, EICs of [M+H-H_2_O] ^+^at *m/z* 379.33593, [M+H-H_2_O] ^+^ at *m/z* 395.33084 and [M+H-H_2_O] ^+^ at *m/z* 411.32576, and [M+H-H_2_O] ^+^ at *m/z* 381.31519 for VitD_2_, 25(OH) D_2_ and 1,25(OH)_2_ D_2_ and IS, respectively.

**Method validation**

The developed method was validated for the determination of the vitamin D and its metabolites in control and disease patient. The specificity of the method was investigated for VitD_3_, VitD_2_ and its metabolites by screening analysis of stripped blank human serum and spiked serum samples. Two other blank plasma samples containing an IS concentration of 50.0 ng/mL were also prepared and tested for interference. Each blank sample was tested for omission of endogenous interference at the retention times of analytes and IS. The chromatograms showed no significant interference around the retention times of analytes and IS in analyte free human serum samples.

Due to high sensitivity of LC/APCI/HRMS, even small amount of sample carryover could cause a positive bias hence needle cleaning procedure, post run column wash was included. No detectable carryover was observed in frequent analysis of blank samples immediately after an injection of QC samples

The linearity of the method was evaluated by using six calibration standards over a calibration range of 3-200 ng/mL for VitD_3_, 25(OH)D_3_ and VitD_2_ and 5-200 ng/mL for 1,25(OH)_2_ D_3_ and 25(OH)D_2_ and 1, 25(OH)_2_ D_2_ in human serum. The calibration data were analyzed by linear least-square regression analysis. The calibration curve exhibited a good linearity between peak area ratios and the concentrations of analytes with a mean correlation coefficient, r^2^ of greater than 0.9995 for all the analytes. The limit of detection for VitD_3_, 25(OH) D_3_ and for 1, 25(OH) _2_ D_3_ was 0.8 ng/mL, 0.8ng/mL and 2ng/mL, respectively whereas for VitD_2_, 25(OH) D_2_ and for 1, 25(OH)_2_ D_2_ was 0.8 ng/mL, 0.8ng/mL and 2ng/mL, respectively. The limit of quantification for VitD_3_, 25OHD_3_ and VitD_2_ was 3ng/mL and 1, 25(OH)_2_ D_3_ and 25(OH)D_2_ and 1, 25(OH)_2_ D_2_ was 5ng/mL. Intra- and inter-batch precision and accuracy of the developed method were investigated by analyzing QC samples at three different concentrations for six replicates including lower limit of quantification concentration. The precision of the method was determined by % CV and accuracy was evaluated by recovery. The intra and inter day precision of VitD_3_, 25(OH) D_3_ and 1, 25(OH) _2_ D_3_ were ranged from 1.13% to 3.81%, 1.45% to 3.89% and 0.62% to 5.04%, respectively. Whereas, intra and inter day precision VitD_2_, 25(OH) D2 and 1, 25(OH)_2_ D_2_ 0.97% to1.39%, 1.69% to 4.09% and 1.17% to 6.35%, respectively. Accuracy for VitD_3_ and its metabolites was > 89% whereas for VitD_2_ and its metabolites was > 91%. Matrix effect was assessed at three QC samples of different concentration for six replicates. To determine matrix effect blank stripped serum samples were processed and then analytes and IS were spiked to the post processed sample. Further, aqueous solutions of analytes of the same concentrations were prepared and analyzed. No matrix effect was observed.

Stability of VitD_3_, VitD_2_, and its metabolites were assessed for auto sampler stability, ambient temperature, long term storage and three freeze thaw cycle. It is evident that all analytes were stable at 4°C over 24 h and at -80°C for 30 days. The Vitamin D and it metabolites were also stable at for 3 freeze-thaw cycles whereas it was unstable at ambient temperature.

A robust analytical method in clinical application is highly desirable because of the large number of samples and variation in the concentration of analytes and matrices among patients. One of the major primary concerns of robustness study was whether the developed UPLC/APCI/HRMS method is sufficiently robust for a large-scale clinical investigation. Therefore, an extensive assessment of the robustness of the quantitative method was conducted. The robustness of the method was evaluated after every 45 injections. Two QC (LQC and MQC) concentration were analyzed for every 45 injections for total six times (*n=6*). The reproducibility of calculated concentrations, retention times, and peak shapes of all analytes were considered for robustness of the study. The % RSD for all the calculated concentration of vitamin D and its metabolites was in the range of 2-10% whereas for retention times it was 0.6%-1.8%. VitD_3_, VitD_2_ and its metabolites have peak symmetry and good sensitivity.

350

360

370

380

390

400

410

420

m/z

0

10

20

30

40

50

60

70

80

90

100

385.34482

C

27

H

45

O

=

385.34649

-4.33378 ppm

367.33437

C

27

H

43

=

367.33593

-4.24681 ppm

0.0

0.5

1.0

1.5

2.0

2.5

3.0

3.5

4.0

4.5

5.0

Time (min)

0

20

40

60

80

100

NL:

4.68E3

m/z=

385.34-

385.34 MS

me-d3-10ng

NL:

1.70E4

m/z=

385.34-

385.34 MS

me-d3-40ng

NL:

6.54E5

m/z=

385.34-

385.34 MS

ME-D3-

200ng

**Figure S 1**: Extracted ion chromatogram and positive ion APCI/HRMS spectra of Vitamin D_3_

0.0

0.5

1.0

1.5

2.0

2.5

3.0

3.5

4.0

4.5

5.0

Time (min)

0

20

40

60

80

100

NL:

1.28E3

m/z=

383.33-

383.33 MS

me-d3-10ng

NL:

4.52E4

m/z=

383.33-

383.33 MS

me-d3-40ng

NL:

1.74E5

m/z=

383.33-

383.33 MS

ME-D3-

200ng

350

360

370

380

390

400

410

420

m/z

0

10

20

30

40

50

60

70

80

90

100

383.32942

C

27

H

43

O

=

383.33084

-3.70439 ppm

365.31896

C

27

H

41

=

365.32028

-3.61328 ppm

**Figure S2**: Extracted ion chromatogram and positive ion APCI/HRMS spectra of Vitamin 25 (OH) D_3_

0.0

0.5

1.0

1.5

2.0

2.5

3.0

3.5

4.0

4.5

5.0

Time (min)

0

20

40

60

80

100

NL:

1.86E3

m/z=

381.31-

381.31 MS

me-d3-10ng

NL:

6.64E3

m/z=

381.31-

381.31 MS

me-d3-40ng

NL:

1.40E4

m/z=

381.31-

381.31 MS

ME-D3-

200ng

350

360

370

380

390

400

410

420

m/z

0

10

20

30

40

50

60

70

80

90

100

381.31358

C

27

H

41

O

=

381.31519

-4.22225 ppm

399.319333

C

27

H

43

O

2

=

399.32576

-4.50767 ppm

363.30314

C

27

H

39

=

363.30463

-4.10126 ppm

**Figure S3**: Extracted ion chromatogram and positive ion APCI/HRMS spectra of Vitamin 1, 25 (OH)_2_ D_3_

0.0

0.5

1.0

1.5

2.0

2.5

3.0

3.5

4.0

4.5

5.0

Time (min)

0

20

40

60

80

100

NL:

3.95E3

m/z=

379.33-

379.33 MS

me-d2-10ng

NL:

1.46E4

m/z=

379.33-

379.33 MS

me-d2-40ng

NL:

1.63E5

m/z=

379.33-

379.33 MS

ME-D2-

200ng

330

340

350

360

370

380

390

400

410

420

m/z

0

10

20

30

40

50

60

70

80

90

100

379.33026

C

28

H

43

=

379.33593

-3.71708 ppm

**Figure S4**: Extracted ion chromatogram and positive ion APCI/HRMS spectra of Vitamin D_2_

0.0

0.5

1.0

1.5

2.0

2.5

3.0

3.5

4.0

4.5

5.0

Time (min)

0

20

40

60

80

100

NL:

9.03E3

m/z=

395.33-

395.33 MS

me-d2-10ng

NL:

2.10E4

m/z=

395.33-

395.33 MS

me-d2-40ng

NL:

1.14E5

m/z=

395.33-

395.33 MS

ME-D2-

200ng

330

340

350

360

370

380

390

400

410

420

m/z

0

10

20

30

40

50

60

70

80

90

100

395.32467

C

28

H

43

O

=

395.33084

-3.51610 ppm

377.31903

C

28

H

41

=

377.32028

-3.31285 ppm

**Figure S5**: Extracted ion chromatogram and positive ion APCI/HRMS spectra of Vitamin 25(OH) D_2_

0.0

0.5

1.0

1.5

2.0

2.5

3.0

3.5

4.0

4.5

5.0

Time (min)

0

20

40

60

80

100

NL:

1.34E3

m/z=

393.31-

393.31 MS

me-d2-10ng

NL:

6.77E3

m/z=

393.31-

393.31 MS

me-d2-40ng

NL:

5.22E4

m/z=

393.31-

393.31 MS

ME-D2-

200ng

360

370

380

390

400

410

420

430

m/z

0

10

20

30

40

50

60

70

80

90

100

393.30901

C

28

H

41

O

=

393.31519

-4.11890 ppm

411.32395

C

28

H

43

O

2

=

411.32576

-4.40042 ppm

375.30305

C

28

H

39

=

375.30463

-4.20993 ppm

**Figure S6**: Extracted ion chromatogram and positive ion APCI/HRMS spectra of Vitamin 1, 25 (OH)_2_ D_2_

0.0

0.5

1.0

1.5

2.0

2.5

3.0

3.5

4.0

4.5

5.0

Time (min)

0

20

40

60

80

100

40

50

60

70

80

90

100

NL:

3.55E2

m/z=

234.35-

235.35 MS

ME-D2-

200ng

NL:

1.36E4

m/z=

381.35-

381.35 MS

ME-D2-

200ng

330

340

350

360

370

380

390

400

410

420

m/z

0

10

20

30

40

50

60

70

80

90

100

381.35009

C

28

H

45

=

381.35158

-3.90717 ppm

399.36057

C

28

H

47

O

=

399.36214

-3.93128 ppm

**Figure S7**: Extracted ion chromatograms of blank, zero sample and positive ion APCI/HRMS spectra of dihydrotachysterol (DHTS)

**Table S1: Determination (n=6) of intra-and inter-day precision and accuracy**

| **Analytes** |  | **Intra-day precision and accuracy** | | | **Inter-day precision and accuracy** | | |
| --- | --- | --- | --- | --- | --- | --- | --- |
|  | **QC Sample** | **LQC** | **MQC** | **HQC** | **LQC** | **MQC** | **HQC** |
|  | **Theoretical concentration (ng/mL)** | 3 | 10 | 200 | 3 | 10 | 200 |
| **Vit D_3_** | **Mean estimated concentration (ng/mL)± SD** | 2.72 ±0.08 | 9.48 ±0.27 | 193.45 ± 3.91 | 2.71±0.09 | 8.96±0.34 | 183.96±2.08 |
|  | **Precision (CV, %)** | 2.98 | 2.91 | 2.02 | 3.66 | 3.81 | 1.13 |
|  | **Accuracy (%)** | 91.22 | 94.85 | 96.72 | 90.59 | 89.69 | 91.98 |
| **25 (OH) Vit D_3_** | **Mean estimated concentration (ng/mL)± SD** | 2.74±0.10 | 9.53±0.19 | 190.12±7.30 | 2.86±0.07 | 9.00±0.34 | 186.49±2.71 |
|  | **Precision (CV, %)** | 3.89 | 2.07 | 3.84 | 2.49 | 3.80 | 1.45 |
|  | **Accuracy (%)** | 90.56 | 95.06 | 95.06 | 95.45 | 90.00 | 93.24 |
| **1,25 (OH)_2_ Vit D_3_** | **Theoretical concentration (ng/mL)** | 5 | 10 | 200 | 5 | 10 | 200 |
|  | **Mean estimated concentration (ng/mL)± SD** | 4.35 ± 0.03 | 9.56±0.26 | 187.94±1.18 | 4.55±0.22 | 9.72±0.11 | 187.02±1.16 |
|  | **Precision (CV, %)** | 0.78 | 2.77 | 0.62 | 5.04 | 1.16 | 0.62 |
|  | **Accuracy (%)** | 87.22 | 95.61 | 93.97 | 89.90 | 97.25 | 93.51 |
| **Vit D_2_** | **Theoretical concentration (ng/mL)** | 3 | 10 | 200 | 3 | 10 | 200 |
|  | **Mean estimated concentration (ng/mL)± SD** | 2.83±0.08 | 9.73±0.15 | 192.41±2.53 | 2.88±0.04 | 9.75±0.09 | 194.01±2.21 |
|  | **Precision (CV, %)** | 3.09 | 1.61 | 1.31 | 1.45 | 0.97 | 1.14 |
|  | **Accuracy (%)** | 94.82 | 97.33 | 96.20 | 96.20 | 97.59 | 97.00 |
| **25 (OH) Vit D_2_** | **Theoretical concentration (ng/mL)** | 5 | 10 | 200 | 5 | 10 | 200 |
|  | **Mean estimated concentration (ng/mL)± SD** | 4.72±0.08 | 9.25±0.15 | 191.49±3.37 | 4.52±0.14 | 9.26±0.17 | 186.69±7.64 |
|  | **Precision (CV, %)** | 1.83 | 1.69 | 1.76 | 3.28 | 1.84 | 4.09 |
|  | **Accuracy (%)** | 93.99 | 92.51 | 95.74 | 90.52 | 92.61 | 93.34 |
| **1,25 (OH)_2_ Vit D_2_** | **Mean estimated concentration (ng/mL)± SD** | 4.53±0.24 | 9.28±0.28 | 191.66±2.25 | 4.56±0.24 | 9.10±0.57 | 192.61±6.61 |
|  | **Precision (CV, %)** | 5.43 | 3.02 | 1.17 | 5.41 | 6.35 | 3.43 |
|  | **Accuracy (%)** | 92.07 | 92.80 | 95.83 | 91.31 | 91.03 | 96.30 |

**Table S2: Spearman correlation analysis of vitamin D metabolites with the diabetes and cardiovascular risk factors**

| **Variables** | **Vitamin D3** | | **25 OH D3** | | **1,25 (OH) D3** | | **Vitamin D2** | | **25 (OH) D2** | | **1,25 (OH)D2** | | **Total 25 (OH) D** | | **Total 1,25 (OH) D** | |
| --- | --- | --- | --- | --- | --- | --- | --- | --- | --- | --- | --- | --- | --- | --- | --- | --- |
|  | r | P value | r | P value | r | P value | r | p | r | P value | r | p | r | P value | r | P value |
| **Age** | -0.10386 | 0.1618 | -0.05749 | 0.4395 | -0.13661 | 0.0652 | 0.13380 | 0.0710 | 0.03754 | 0.6139 | -0.09057 | 0.2227 | -0.14661 | 0.0621 | -0.11516 | 0.1206 |
| **Gender** | **0.18223** | **0.0135** | 0.06082 | 0.4134 | **0.14764** | **0.0461** | **-0.16171** | **0.0287** | 0.01675 | 0.8220 | 0.03768 | 0.6126 | -0.13767 | 0.3455 | 0.10665 | 0.1507 |
| **BMI** | 0.06304 | 0.3966 | -0.02876 | 0.6992 | 0.09947 | 0.1804 | 0.09762 | 0.1886 | -0.02266 | 0.7608 | 0.08521 | 0.2514 | 0.24437 | 0.0906 | 0.10100 | 0.1737 |
| **HBA1c (%)** | -0.05555 | 0.4551 | **-0.25002** | **0.0006** | **-0.16219** | **0.0283** | 0.03650 | 0.6237 | -0.06587 | 0.3757 | -0.11073 | 0.1356 | **-0.26302** | **0.0004** | **-0.14955** | **0.0433** |
| **FBS (mg/dl)** | -0.10540 | 0.1556 | **-0.21637** | **0.0033** | **-0.14855** | **0.0448** | -0.00928 | 0.9008 | -0.12932 | 0.0810 | -0.09491 | 0.2013 | **-0.21366** | **0.0041** | -0.13288 | 0.0729 |
| **Systolic BP (mmHg)** | 0.05327 | 0.4739 | 0.01528 | 0.8374 | -0.01766 | 0.8124 | 0.08553 | 0.2496 | -0.00049 | 0.9948 | 0.11837 | 0.1105 | -0.23464 | 0.1046 | 0.06215 | 0.4033 |
| **Diastolic BP (mmHg)** | -0.03889 | 0.6012 | -0.06535 | 0.3795 | -0.09662 | 0.1932 | 0.04109 | 0.5808 | -0.05099 | 0.4930 | 0.01659 | 0.8237 | **0.31827** | **0.0258** | -0.04808 | 0.5181 |
| **Creatinine (mg/dl)** | **-0.20135** | **0.0063** | -0.04714 | 0.5263 | -0.08169 | 0.2716 | 0.01503 | 0.8400 | -0.12543 | 0.0907 | -0.01778 | 0.8112 | **0.31650** | **0.0267** | -0.06003 | 0.4195 |
| **eGFR (ml/min/1.72 m^2^)** | **0.14641** | **0.0480** | 0.02002 | 0.7880 | 0.06216 | 0.4032 | -0.02006 | 0.7875 | 0.07597 | 0.3067 | 0.00680 | 0.9272 | **-0.33909** | **0.0172** | 0.04303 | 0.5630 |
| **Uric acid (mg/dl)** | 0.04074 | 0.5839 | 0.18861 | 0.0106 | 0.08402 | 0.2582 | 0.01645 | 0.8250 | -0.07705 | 0.2999 | 0.13353 | 0.0715 | **0.27055** | **0.0601** | 0.12284 | 0.0976 |
| **CK_MB (u/l)** | -0.14312 | 0.0533 | -0.06949 | 0.3500 | -0.08037 | 0.2794 | **-0.17345** | **0.0189** | 0.00116 | 0.9876 | -0.08795 | 0.2365 | 0.01470 | 0.9202 | -0.08619 | 0.2460 |
